# The co-leadership dance: the building of a managerial we in functionally shared leadership in healthcare

**DOI:** 10.1108/LHS-11-2024-0134

**Published:** 2025-11-21

**Authors:** Marianne Döös, Linda Sturesson Stabel, Mia von Knorring

**Affiliations:** Department of Education, Stockholm University, Stockholm, Sweden; Department of Learning, Informatics, Management and Ethics, Karolinska Institutet, Stockholm, Sweden

**Keywords:** Management effectiveness, Qualitative research, Health care, Doctors, Leaders,, Line management

## Abstract

**Purpose:**

Changing healthcare organizations call for more collective and team-based approaches to leadership. This study focuses urgent care units in Sweden where unit managers and physicians responsible for medical management are expected to co-lead as equals in the organizational hierarchy in a form conceptualized as functionally shared leadership (FSL). This study aims to increase the current scientifically based knowledge about putting FSL into practice.

**Design/methodology/approach:**

Data was collected through interviews on two occasions with individuals in couples leading six publicly driven urgent care units, and through conversations with the department manager of these units. Data was analyzed in two steps, first with individuals and second with pairs as the basis of analysis. In step 1, an iterative, thematic approach was used. In step 2, the theoretical points of departure served as a lens to direct our focus.

**Findings:**

Amid handling ordinary work tasks and new challenging situations, the couples worked to shape a shared leadership space. Three intertwined activities were identified: working with boundaries, working with structures and relating to each other. The presence of a shared purpose emerged as the glue of co-leading. Metaphorically the couples perform a co-leadership dance where the steps of either influence the other.

**Originality/value:**

The study contributes knowledge relevant for healthcare contexts as well as to the research field of managerial shared leadership concerning cross-functional sharing. It adds understanding of previously understudied informal and processual aspects. The shaping of a shared leadership space is not a separate activity with ready-made mandates.

## Introduction

Changing healthcare organizations call for more collective and team-based approaches to leadership (De Brún *et al.*, 2019) including solutions based on nurse-physician managerial couples (Clausen *et al.*, 2017; Saxena *et al.*, 2018; Thude *et al.*, 2019). Such ways of organizing are increasing (Saxena, 2021) and have previously been studied across a broad variety of healthcare settings and at different organizational levels, e.g. in hospitals (Clausen *et al.*, 2017; Kim *et al.*, 2014; Saxena *et al.*, 2018; Thude *et al.*, 2019), psychiatric care (Steinert *et al.*, 2006), integrated care (Aufegger *et al.*, 2020; Klinga, 2024) and health centers (Gibeau, 2025).

Previous studies show that these ways of organizing healthcare enhance collaborative problem solving and responsibility (Aufegger *et al.*, 2020; Clausen *et al.*, 2017; Saxena *et al.*, 2018) and help bridge competing demands and professional silos (Gibeau *et al.*, 2020; Gibeau, 2025). Potential challenges to its success, such as role ambiguity and lack of organizational support (Clausen *et al.*, 2017; Saxena *et al.*, 2018; Gibeau, 2025) as well as tensions within the leadership constellations and lacking guidance about how to enact the sharing (Mitra *et al.*, 2019) have also been identified.

To further advance the field and increase the usefulness for healthcare practitioners, more studies are needed, especially when it comes to the processual and informal aspects of how this form of co-leadership is established within the sharing couples (Gibeau, 2025; Gibeau *et al.*, 2020; Döös and Wilhelmson, 2021b). In this study, we focus this gap, using data from urgent care units in Sweden where unit managers (mostly nurses) and physicians responsible for medical management are expected to co-lead as equals in the organizational hierarchy.

## Theoretical points of departure

Plurality in leadership is practiced in various forms and sectors with the commonality of going beyond traditional ways of organizing for singularity (e.g. Denis *et al.*, 2012; Clouser *et al.*, 2020). Our theoretical point of departure lies with managerial shared leadership (MSL). The specific form examined is functionally shared leadership (FSL) (see below), and thus represents a co-leadership (Klinga, 2024; Gibeau, 2025) case of cross-functional collaboration (Aufegger *et al.*, 2020) in an institutionalized practice (Gronn, 2002; Janssens *et al.*, 2021).

MSL is here defined as “an organizational phenomenon where a few individuals have and/or take mutual responsibility for the tasks included in holding a managerial position” (Döös and Wilhelmson, 2021b, p. 717), and where leadership tasks and power are distributed within the sharing constellation. What differentiates MSL from other forms of plural leadership is that the sharing takes place in formal managerial functions. Previous research has shown that sharing constellations, to succeed, need to develop a foundation built on common values, trust and lack of pretension (ibid.).

FSL is one of four structural forms of MSL that have been conceptualized by Döös and Wilhelmson (2021b) using the two variables of organizational rank and work task division. These forms (italicized below) are ideal, a kind of map through which formal and informal task division and power can be analyzed. *Joint leadership* is practiced by managers who have the same mandate, assume equal responsibility, share power and accountability,and have merged work tasks. Both report to the same manager, neither is subordinate to the other. Likewise, *FSL* implies formal hierarchical equality and collective responsibility for the managerial whole, but basically with divided work tasks in separate functions. Function here refers to a principle of organizational task division (e.g., Bratton, 2010). Despite basic task division, this form in practice also builds on having a collaborative zone (Döös and Wilhelmson, 2021a). *Horizontally invited leadership* refers to formal equality but implies sharing across organizational unit boundaries. *Vertically invited leadership* implies formal hierarchy of decision-making when it comes to issues of formal responsibility and authority. Despite formal hierarchy, the sharing managers regard each other as equals and practice joint responsibility and joint authority (Heenan and Bennis, 1999).

As our focus is FSL in healthcare, we adopt the definition provided by Mitra *et al.* (2019) where the FSL takes place between a physician and a nonphysician who work as coequals “at the same structural levels within a health care administrative hierarchy and that are supposed to work in partnership to oversee a service with separate, albeit overlapping, responsibilities” (P. 319–320). The work processes in FSL are of specific interest to study as the imposed task separation creates conditions that stand in contrast to other MSL forms.

### The concept of space

To explore the work processes, we have turned to the concept of space. Nonaka and Konno (1998) described the notion of “a shared space for emerging relationships” (p. 40), where learning “takes place in social interaction where explicit and tacit knowledge embedded in organizations meet each other” (Tynjälä, 2008, p. 136). The space concept has previously been developed in relation to MSL studies, albeit from a different role theoretical stance. Gronn and Hamilton (2004) describe role space sharing as a role space normally filled with one appointee that is instead filled by two. Especially, they discuss the dynamics of interdependence, trust, complementarity, overlap and duplication. In studies of FSL couples, trust is defined by symmetrical mutuality and emphasizes both the expectations of another person and the willingness to be vulnerable and to act based on somebody else’s competence (e.g. McAllister, 1995; Wilson and Cunliffe, 2022). Complete or high level trust has been referred to as “a complex intertwining of personal thoughts, feelings and values” (Macmillan *et al.*, 2004, p. 253).

## Previous empirical research on functionally shared leadership (FSL)

In healthcare, FSL has been studied in hierarchically equal and interdisciplinary pair collaborations, for example between a nurse and a physician (e.g. Mitra *et al.*, 2019; Thude *et al.*, 2019), between a physician and a co-leader “with a different background” (Saxena, 2021, p. 137) or between a medical co-director (physician) and an administrative co-director (trained and experienced in management and a non-medical clinical profession) (Gibeau *et al.*, 2020).

It has also been studied in other contexts, especially in performing arts, museums and film industry, where co-leadership between an artistic leader and an administrative leader is the normality (de Voogt, 2006; Ebbers and Wijnberg, 2017; Järvinen *et al.*, 2015; Reid and Karambayya, 2009; Reid and Karambayya, 2016). A common point of departure here, and sometimes in healthcare, is that there is a conflict of logics between roles or tasks built into these couples (e.g. Gibeau *et al.*, 2020; Ebbers and Wijnberg, 2017; Reid and Karambayya, 2009; Reid and Karambayya, 2016). Reid and Karambayya (2016) introduced the concept shadow of history to analyze how past history might influence a relationship. Gibeau and colleagues (2020) found that a common mission existed where the co-leaders spoke about a concern for patients. Finally, a study of putting FSL into practice in schools and pre-schools, identified the necessity of a collaborative zone in the dived mandate (Döös and Wilhelmson, 2021a).

## Aim and research questions

The aim is to increase the current scientifically based knowledge about putting FSL into practice. Specifically, the study contributes knowledge about processual and informal aspects of how this form of co-leadership is established within the sharing couples. Three research questions are posed:

*RQ1*. Which work processes take place when FSL leaders build a shared leadership space?

*RQ2*. How is the FSL collaboration experienced by the sharing couples?

*RQ3*. How can processes and experiences be understood?

The first two questions are empirical and are mainly answered through the findings. The third question concerns prior research and theoretical foundations and is addressed in the discussion.

## Methods

The study has a qualitative approach where cases of FSL between a unit manager (UM) and a physician responsible for medical management (PReMM) are in the center of attention. Data was collected through interviews on two occasions with individuals in couples leading six publicly driven urgent care units and through conversations with the department manager (DM) of these units.

### Setting, study participants and leadership set-up

**Figure 1.**
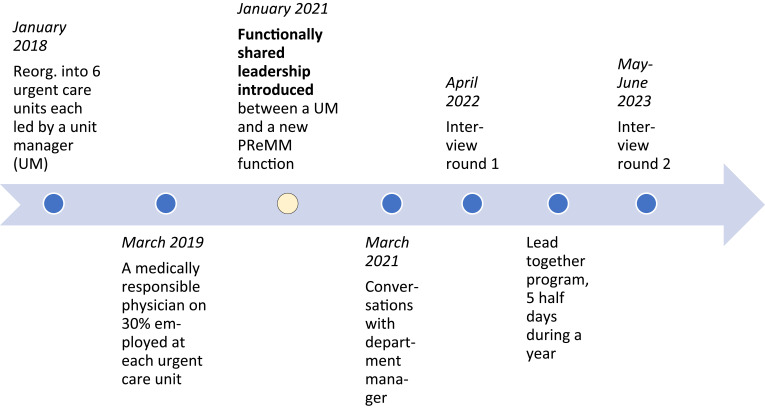
Timeline overview

The emergency care in this healthcare region was reorganized in 2018 (see Figure 1), resulting in the creation of six urgent care units for lesser complicated conditions to relieve the pressure on emergency care. The new units were led by a DM to whom the six UMs reported. Medical responsibilities were organized separately as a senior PReMM led the locally medically responsible physicians of all six units. In January 2021, his tasks were passed downwards to a new local PReMM function of each urgent care unit now reporting to the same DM as the UMs. All six FSL couples were included in the DM’s management team. The DM mandated each couple to lead and develop their urgent care unit together. This meant that an FSL model was implemented, characterized by hierarchical equality in the division of work tasks.

Study participants were identified via their future participation in a program about leading together specifically constructed for the management team on the initiative of the DM. Each couple consisted of a UM, usually a nurse and a PReMM. At the time of the first interviews, the interviewees had formally held their FSL positions for slightly more than a year, except for one couple who had worked together only for a few months. However, the individuals in three couples had been working at the urgent care units since the start 2018, and had some emerging collaboration before the formal FSL start. The UMs were already in place when the PReMMs started in their new managerial function.

The UMs had experience of leadership from training and work before the assignment, whereas the PReMMs in general lacked such experiences. Both were salaried. In their basic assignments the UM had managerial responsibilities over operations, finance, work environment, human resources and staff, whereas the PReMM had responsibility over medical issues and patient safety.

Both worked full-time at the unit, but with differing allocations of time for managerial duties and varying work schedules. The UMs had 100% of their time assigned to managerial tasks and worked standard office hours, whereas the PReMMs worked 70% of their time as clinicians, following a rotating schedule that also included weekends and evening shifts.

### Data collection and analyses

The conversations with the department manager took place in spring 2021 and are here mainly used to contextualize the FSL in terms of assignment and purpose. After a few pilot interviews, the first round of interviews with the 12 people constituting the six sharing couples took place in April 2022. The second round was conducted 14–15 months later with the participation of 11 people. One UM did not answer the interview request. All interviews were conducted by the second author via a digital platform, lasted between 30 and 60 min, were audio-recorded and transcribed *verbatim*. An interview guide was developed by the second and third author. The third author developed and later delivered the Leading together program. We believe that this might have influenced in the direction of increased collaboration between the co-leaders.

The data analysis was conducted in two steps: first, using individual interviews as the basis, and second, focusing on pairs. In the first step, the round 1 interviews were analyzed with an emphasis on the work processes involved in how FSL leaders construct a shared leadership space. The interviews were analyzed using a reflexive thematic analysis approach (Braun and Clarke, 2021). The final themes (activities) and sub-themes (sub-activities) were defined and named as presented in the findings. In the second step, interviews from both rounds 1 and 2 were analyzed using a within-pair approach, focusing on whether the incumbents expressed concordant views on their collaborative experience. This analysis was guided by our theoretical framework, which served as a lens to direct our focus. Each pair’s interviews were read sequentially – first the UM, then the PReMM – while collecting quotes and taking notes on how they described one another and their shared collaboration.

For ethical reasons, the findings are presented at an overall level with anonymized participants. Each participant was assigned a letter from A to L. The couples were assigned a letter from M to R. The quotes have been slightly edited for readability and anonymity and are used as illustrations. All quotes are translated from Swedish; […] is used to indicate omissions.

In line with the Declaration of Helsinki (World Medical Association, 2013), all participants were informed about the aim of the study, procedures and methods used, that data were handled confidentially and could only be accessed by the researchers, that participation was voluntary and that they could withdraw from the study at any time without having to state any reason. All participants gave their oral consent to participate in the study.

## Findings

Below, the activities identified in the shaping of a shared leadership space, during approximately the first year of FSL, are presented. The presentation then turns to how the sharing couples experienced their collaboration within pairs and over time.

### Activities shaping the shared leadership space

**Figure 2.**
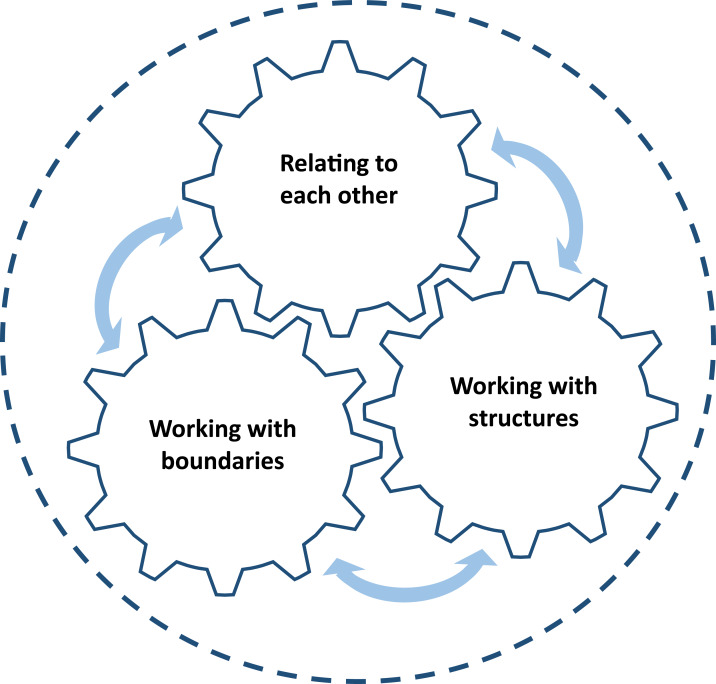
Illustration of three activities used in daily work to shape the shared leadership space during the first year in FSL

Amid handling ordinary work tasks, the couples had worked, and sometimes struggled, to shape their shared leadership space through various activities. Figure 2 illustrates the three activities that were identified in the analysis, each with subsequent sub-activities presented below. The cogwheels illustrate the interrelatedness of the activities, as they were not performed separately but intertwined and part of daily work.

#### Working with boundaries.

The co-leaders were continuously working with task boundaries. They created boundaries between each other within their shared space, explored how these boundaries were drawn and tried to create boundaries around their shared leadership space. Three sub-activities were identified.

In *comparing and contrasting,* they compared their own leadership couple with the couples at the other units, and with the peers who had the same function in other units. They contrasted their own function and responsibility to that of their leadership partner and tried to figure out if their own boundaries were correctly drawn. Especially among the PReMMs, there were examples where they seemed to struggle with how to interpret what leading staff meant, and whether this was within one’s own boundary or not:

I’ve heard, and maybe that’s what stresses me a bit, that in other units the setup is a little different and the PReMM is more involved in personnel issues. I don’t do that, and I don’t perceive that I’m expected to either. (G, PReMM).

The boundaries were recurringly *clarified* by emphasizing the different areas of responsibility within the couple. In addition, the boundaries were clarified and manifested in relation to staff:

If my staff approaches me with things that should be discussed with the PReMM […] I usually clarify that this is the PReMM’s area of responsibility and decision. (D, UM).

Workplace meetings with staff were occasions where leadership boundaries were clarified and manifested. At times, however the UMs seemed to keep a boundary around his/her own leadership space at these meetings, whereas the PReMM was only assigned one of many bullets on the meeting agendas, illustrating that UMs took ownership over meetings instead of them both leading the meetings jointly.

The shared leadership space was also shaped by *crossing* boundaries. This was done in two ways – invited or uninvited. The co-leaders invited each other into their respective areas of responsibility by involving the other. To be involved in each other’s tasks was described as a winning concept. This could be done through providing the other with information on current issues to keep updated about what was going on, through inviting the other to solve problems together, aiming to agree in matters, or to get input and progress in tasks. By involving each other, they developed understanding about the other’s function and area of responsibility. Involving the other was also about standing united and feeling confident and well-informed in relation to staff, which also made it easier to address any questions and concerns.

When no explicit boundaries were drawn or when there was a perceived unclarity or vagueness, for example about who should handle a task, they often discussed, and thereby metaphorically decided where to draw the line – either between them, separately solving the task or around them thereby solving the task together:

Is it a manager who should answer this or should I, as PReMM, do it? Usually it’s clear, but not always and then we must agree, what feels reasonable. (H, PReMM).

Depending on situation, boundaries that were rather clearly drawn between them were sometimes temporarily moved to solve urgent matters even though it was formally the responsibility of one part in the couple. However, crossing boundaries of responsibility and expertise occasionally happened uninvited and was then experienced as problematic by the one who’s area had been stepped into. This uninvited boundary crossing created frustration and uncertainty within the couple and could lead to confusion amongst staff concerning who was responsible and accountable. When someone had crossed a boundary uninvited, it was emphasized that the sharing couple had to sit down and talk to each other to solve the situation.

There were examples when the UMs wanted the PReMMs to be more involved across boundaries, but at the same time, kept the boundaries through not inviting the other. However, we as well found that PReMMs were sometimes described to step back from issues where they were supposed to be involved, such as finance or leadership.

The above work with boundaries was constantly ongoing for the couples and took place in dialogues where the couple discussed, mutually reflected and solved problems together. These boundary-related dialogues were described as necessary.

#### Working with structures.

To manage operations jointly, the co-leaders emphasized the importance of establishing structures that would support their collaboration. Three sub-activities were identified.

*Finding forms to meet* had sometimes been a struggle for the couples. The sharing couple had to decide when and how often they would meet, for how long, if any other functions were to be included and the meeting content. The meetings had to be perceived as meaningful, for the structure to be maintained. Some had tried to organize a structure of scheduled meetings on a regular basis, but these often tended to change, and sometimes even disappear. The co-leaders had difficulties in shielding themselves and their scheduled time and chose to deal with issues immediately instead. However, despite the struggle with finding a structure for meetings, when at the workplace simultaneously the couples took time to meet. These informal meetings appeared spontaneously, regularly and face-to-face, and for some couples, these had become a habit of daily reconciliation during the morning or afternoon coffee or before ending the workday:

If I’m busy or sitting in my office – my office is a little further up the corridor – the PReMM usually texts me. “Time for a cup of coffee, exchange a few words?” Or the PReMM takes his coffee and comes to me, and we sit together and discuss the case or issue. (B, UM).

They had contact via phone, email and text messages, but not being able to meet face-to face during longer periods was described as challenging. Some couples often kept in touch during spare time.

Physical proximity was perceived as enabling close collaboration, spontaneous meetings, as well as urgent discussions for problem-solving. However, physical proximity could also lead to interruptions if a UM stepped in and interrupted the PReMM in clinical work to deal with a matter directly.

In the process of *building working methods*, structures were partly formed by making comparisons with the other units but were also based on the leaders’ experiences from previous workplaces. Similar experiences were emphasized as making their working methods and thinking aligned while differences required more talking to agree.

Potential differences in approaches and ways of working were either not attended to with reference to the work situation at the unit or were handled in that the leadership couple had frequent conversations trying to coordinate views to agree upon matters and have mutual ideas about the goal scenario. This was described as specifically important before meeting others, whether individual co-workers or the whole staff, to ensure agreement and to prevent the staff from playing them off against each other:

If we are going to sit down and talk to the employees about how we want things to be organized, then we also need to feel confident that we have the same view and the same vision going forward. (L, PReMM).

There were recurrent expressions of difficulties in finding occasions to discuss more long-term issues and *finding ways forward.* UMs and PReMMs expressed a need to discuss and mutually reflect on the unit’s purpose and goals and how to improve and develop the unit. Reflecting together on overall and long-term issues was however rarely done due to perceived lack of time and a constant need to solve more urgent situations arising from the daily work.

#### Relating to each other.

Finally, a shared leadership space was shaped by the co-leaders relating to each other. Three sub-activities were identified.

Here the interviewees described how they were *getting to know and care for each other* in terms of how they, within their couple, had developed “a friendship”, had “chemistry” or had “found” each other. They highlighted positive qualities such as their co-leader being calm, responsive, flexible, non-pretentious, or straight forward. They also mentioned both having similar qualities or that the other complemented one’s own lack of the same. Through the process of getting to know each other, a feeling of trust and safety developed.

There was in some cases some discrepancy in the sharing couples on how well they thought they related. Feelings of unease and frustration and expectations which were not met by the other sometimes occurred, which made them, have to *deal and cope with each other.* Unpleasant feelings and unmet expectations mostly related to the other’s behavior in different situations:

Sometimes it happens that issues are raised at the wrong time or in the wrong forum, and then you [suddenly] realize something has been decided. So, there are small things that we need to change in our way of working (I, PReMM).

The co-leaders emphasized how they complemented each other and how they could *take advantage of each other’s qualities*. The UM became the PReMM’s channel to knowledge about the organization, and to the overall perspective. In turn, UMs could ask for input from the PReMMs when employing new physicians as the PReMMs knew what medical skills was needed. In this respect, the PReMMs acted as bridges between themselves and the physicians, a group that UMs sometimes had difficulties to handle as a manager:

I have not been the head of physicians before. […] I have perhaps been extremely careful in how I have progressed in that group […]. It hasn’t been easy. […] you must really prove that you are able to lead for this group. (C, UM).

The above work with relating to each other largely took place as integrated in working with boundaries and structures.

### The FSL collaboration as experienced by the co-leaders as couples

Leaving the level of concrete doings in the activities described above, our attention now turns into the narrative of how the sharing couples experienced their collaboration. This findings section combines data from interviews conducted slightly more than a year after the formal introduction of FSL (round 1) with data from interviews made a little more than a year later (round 2) (see Figure 1 in Methods for the timeline).

#### Reflections on the sharing collaboration.

The round 1 interviews contain numerous comments and reflections that characterize the perceived quality of the FSL collaboration. To exemplify wordings such as “a good relationship”, “very secure”, “ideas being exchanged”, “no prestige”, “a very close collaboration”, “mutual trust”, “listen to each other” and “same sense of humor” were used. Table 1 illustrates the within-couple way of talking about the collaboration quality. The first example (R) describes a non-problematic experience, whereas the second example (Q) shows that the PReMM is more hesitant in the experience of the collaboration.

**Table 1.** Two within-couple examples of talk about the collaboration quality

The UM	The PReMM	Couple
Very close collaboration	Work very closely	R
Listen to each other	Same outlook	
100% trust	Pretty good routines regarding task division	
Really enjoy working together	Both pretty quick to make decisions	
Incredibly good relationship	Dancing around each other a bit	Q
Pretty in sync	UM has never been unfair	
Found each other quickly	Could have better communication	

In round 1, some co-leaders mentioned changes and improvements they hoped to see as a natural part of their collaborative development. While some wanted to meet more often and to structure and synchronize their time better, others mentioned that it would be good for the UM to step back and for the PReMM to step up to be more visible as a leader in relation to staff. Other examples were about finding structure and longtime goals, to learn how to make people (staff) collaborate, to involve the PReMM more in the whole or in economic issues or to make the voice of the physician group more heard. A little over a year later, in interview round 2, the co-leaders were explicitly asked whether they believed a UM and a PReMM should lead together. Consistently, with a single exception of a PReMM who did not find the model sufficiently equal, the couples had confidence in the model. Thus, overall, the FSL model was endorsed. Furthermore, that most PReMMs from the beginning were not fully equipped for the task had now moved to the better. Overall, the PReMMs had grown into the co-leadership function.

Analysis of the interviews made it possible to assess concordance as a within-pair quality for each couple. The following two quotes illustrate how both parties tell a similar story, indicating concordance:

I involve the UM in matters that are purely medical, which is my table, and the UM involves me, or at least keeps me informed, about things that do not have a medical value at all. That’s good because then you understand the other role a little better. (PReMM, P).

The PReMM has invited me into the patient safety part, involved me in the cases and anchored with me – is it okay like this? What do you think? I want your input. (UM, P).

The first round of interviews showed concordance for four of the couples, whereas two told somewhat differing stories, although not conflicting with one another (see Table 2). A year later, there appeared to be a shift toward greater concordance. The four couples (M, O-Q) possible to assess in round 2 either continued to be concordant in their narratives or had moved from differing to concordant or less differing. In between the interview rounds the PReMM of couple N had changed jobs.

**Table 2.** The couples’ talk about the collaboration

Talking about the collaboration in interview round 1	Talking about the collaboration in interview round 2	Couple
Concordant	Concordant	M
Concordant	_–_^a)^	N
Differing although not in conflict	Less differing, more concordant	O
Concordant	Concordant	P
Differing although not in conflict	Concordant	Q
Concordant	_–_^b)^	R

Note(s):

^a)^No longer a couple. ^b)^The UM was not interviewed

#### Reflections on the functionally shared leadership model.

An important aspect concerns the explanations of why the model had potential to function well. Besides recognizing that nobody could carry the responsibility alone, the strength of having different professional backgrounds was emphasized, as it enabled contributions from diverse perspectives. These different perspectives were described in various ways, such as budget versus medicine, or nursing versus medicine:

An operation like this needs a manager and also someone with medical responsibility. I believe it’s an advantage if these are not the same person, because sometimes there can be interests in conflict with each other […] there’s strength in being two. (PReMM, P).

It was also stressed that healthcare’s common mission and ways of working facilitated the collaboration despite potential conflicting interests and professional hierarchies:

In healthcare, there are well-established ways of working […] there is a shared goal in healthcare that has always been at the core […] it’s quite similar in healthcare everywhere, even internationally, because it’s the same task that needs to be solved all the time. […] There are structures that run deep. (PReMM, R).

#### The crafting of better conditions.

The interviews in round 2 showed that the couples had reached a level of crafting better conditions for leading together. Several improvements were of an organizational nature. For example, local leadership teams had been established, schedules had been synchronized to ensure shared presence, PReMM’s admin time had been moved to a day on-site at the urgent care center, the couples had allocated at least one day a week for joint presence and rearranged their calendars to meet more frequently. Furthermore, meetings were described as more structured where time is set aside, they reflect, take minutes and follow up. In other words, these are not just meetings *per se*, but meetings within a system they know how to use and understand the purpose of.

Improvements were often concrete, such as a PReMM being provided with a workspace, including a personal desk and computer, in the same room as the UM. Or, in another case, a UM who tended to dominate joint staff meetings chose not to attend certain meetings. This allowed responsibility and trust to be transferred, making the PReMM more visible as a leader.

#### The work task dance metaphor.

Metaphorically speaking, the FSL journey has led the couples seem to perform a work-task dance – not a solo, but a couple dance, where the steps of either influences the other. This dance is grounded in the three activities related to boundaries, structures and relationships, where an ongoing give-and-take of space shapes how they work. In the interviews this is portrayed both as ideas or wishes for the future and as concrete changes and steps made or in the making. Following the metaphor, there are two directions at play: one where the co-leaders move closer together to conduct tasks jointly, and another that leads in the opposite direction – toward the division of tasks that still needs to exist. The leaders are stepping back and stepping forward, sometimes bowing and adjusting with smaller steps when needed. The pace of this work-task dance seems to vary, speeding up or slowing down depending on the co-leaders and on what is happening in their local context.

Over time, the views on shared leadership in this “dance” showed a shift toward greater joint responsibility within the couple. Two quotes exemplify:

All of this we need to do together […] the grey zone that we thought was this narrow […] ended up being what we would work with. (UM, P).

The unit manager and I have a much more intertwined approach. […] I realize now that one must take on a leadership role. (PReMM, P).

The co-leaders had become aware that several tasks had an ingredient of them both needing to take responsibility, despite only one having formal responsibility. This represents a change in view and is referred to an exercise taking place in their leadership program where the couples once listed individual vs joint tasks. This was visualized as moving post-it notes on a whiteboard. This exercise is often referred to in the interviews and led to changed views concerning where one would step forward or back off in the dance. The work task dance has been ongoing because the FSL start but was during the program restarted or intensified.

Depending on where they are in their collaboration, there can simultaneously be a need for movement in opposite directions within a pair, toward more togetherness, but also toward more separation of other tasks, out of respect for the other’s area. Shared and divided are therefore not entirely opposites but can at times support one another.

## Discussion

This study contributes knowledge about FSL, a specific form of MSL. Specific for this form is the basic division of task, a division that comes with a risk to hamper clarity if being over-emphasized (Döös and Wilhelmson, 2021a). The current study shows how an ongoing work task dance during the first two years of FSL takes place as a move in the direction of viewing tasks as shared, as joint, in a collaborative zone.

Healthcare is a branch where MSL is practiced and also researched, typically as co-leadership between a nurse and a physician (e.g., Thude *et al.*, 2019; Mitra *et al.*, 2019) or between nurse managers (e.g. Rosengren and Bondas, 2010; Döös *et al.*, 2017). The couples in our study were not chosen to represent a research interest in a specific problem in relation to MSL – like competing demands or conflicting organizational logics (Gibeau *et al.*, 2020; Reid and Karambayya, 2016). Our study participants were chosen as they are an example of FSL being put into practice in a regular healthcare system; thus, in contrast both to the mature co-principalship of Gronn and Hamilton (2004) and to organizations where the co-leadership was particularly problematic (Reid and Karambayya, 2009).

### The shaping processes – the building of a managerial we

**Figure 3.**
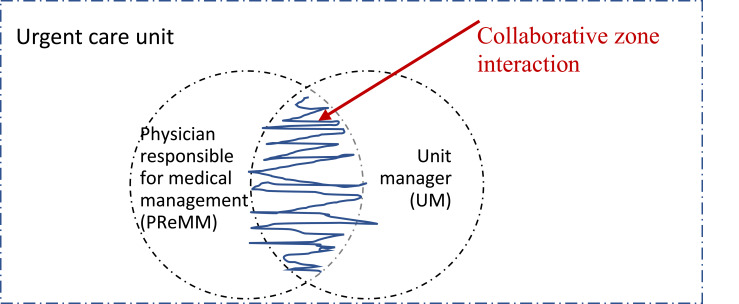
Illustration pointing to the collaborative zone interaction as overlapping the managerial mandates

In assigning FSL from above in an organization it might be easy to believe that mandates and task division are ready-made from the start as clarified in job descriptions. This is not the case and thereby FSL is a challenging model as important issues that must be dealt with cannot be solved by returning to a hierarchical structure if disagreement is at hand. The current study contributes the understanding of how the co-leaders’ shape a shared space for emerging relationships (Nonaka and Konno, 1998), i.e. how they gradually build a managerial we. This was a continuous work-integrated learning process of three activities: working with boundaries, working with structures and relating to each other. The co-leaders were repeatedly and iteratively setting and resetting, constructing, and reconstructing their boundaries, structures and relationships. This supports previous research about FSL in the education sector showing that the key to well-functioning sharing was not the division but the collaboration of the couple (Döös and Wilhelmson, 2021a). Thus, the concept of a collaborative zone was established and understood as the tasks where the sharing leaders need to coordinate, both in their understanding of the tasks and in their practical actions (See Figure 3).

The study reveals that over time the collaborative zone grew, more tasks are so to say placed in the middle as the co-leaders integrate their respective areas. Becoming united developed and maintained the leadership space and, with reference to Nonaka and Konno (1998) and Tynjälä (2008), made it possible to metaphorically enter the space, and by bridging even act with legitimacy in the other’s area of expertise or responsibility.

In the words of Gronn and Hamilton (2004) a role space normally filled with one appointee is instead filled by two people, or in our view by the interactions of two people; to enable conjoint agency (Gronn, 2002) “requires negotiation of sets of norms for articulating the work” (p. 14). Our study shows that the collaboration norms were only to a minor extent pre-specified; interpreting the findings, the norms were emerging through negotiations integrated in each couple’s activities.

Drawing on Wilson and Cunliffe (2022), we argue that it is in the shaping of an open-ended shared leadership space that the couples, in the same breath, create trusting relationships in an iterative and emergent process. Wilson and Cunliffe asserted that trust develops when “both participants increasingly value and respect what each other brings to the relationship” (p. 2). Mutual trust has been described as a *sine qua non* quality in the bedrock of all successful MSL (Döös and Wilhelmson, 2021b) and should not be viewed as an outcome, but as a part of a process of relationship development (Wilson and Cunliffe, 2022).

### Shadows of history

Inspired by the idea of shadows casted from history (Reid and Karambayya, 2016), we here shed light on our findings in two respects, solo leadership vs MSL and conflicting demands vs mission. Before FSL, our organization was unaccustomed to MSL, yet this was not valid for all individuals as a few had such experience, albeit more in the form of joint leadership (i.e. basically having tasks merged and not divided). In terms of values expressed, there seems to be no negative shadow casted from the organizational history of solo leadership. Rather the FSL model was embraced by the co-leaders. Yet, on a closer look, the work task dance descriptions where some unit managers had difficulties to step back could be interpreted as such a shadow. On the other hand, the shadow here casted does not seem to stem from believing in solo leadership. Rather it seems to reflect the habit of being a responsible manager in combination with the circumstance that the UMs were already managers when FSL was introduced and the PReMMs were mandated to co-lead.

Another shadow reflects an established, natural task division between medical and operational aspects, or between medical aspects and nursing. The overall feeling is the absence of conflicts. The co-leaders stress that they need each other. It is even said that it is good to be two *because* there are conflicting interests. Thus, “collective leadership arrangements might bridge competing logics” (Gibeau *et al.*, 2020, p. 470). We argue the importance of mission, identified by Gibeau *et al.* (2020). Mission, or the presence of a shared purpose, emerges as the glue of co-leading and has similarities to the necessity of shared values for well-functioning MSL (Döös and Wilhelmson, 2021b). Furthermore, work task conflicts handled within the couple (Reid and Karambayya, 2009) can be productive when matters are talked through. A disagreement on a special issue does not inevitably turn into a conflict between the co-leaders, even though the matter at hand is difficult.

### The work task dance – a cognitive and emotional exercise

As said above, the couples seem to perform a metaphorical work task dance where the co-leaders move closer together to carry out tasks jointly, or toward the division of tasks that still must exist. The co-leaders had pre-specified areas of responsibility articulated in their mandates, in practice the areas were rather emerging. A formal division is thereby dissolved in informal practice, as the leaders, in constructing their shared leadership space, come to view themselves as equals and engage in joint responsibility and authority (Heenan and Bennis, 1999). This difference between formal and informal is built into the definition of MSL where individuals *have and/or take* mutual responsibility for the tasks included in holding a managerial position (Döös and Wilhelmson, 2021b). Over time it becomes evident that PReMMs, who when starting were not fully equipped to lead, have started to take responsibility also in issues where formal responsibilities lie with UMs. Thus, even within this egalitarian structure with divided responsibilities, we still see informal shifts occurring. The parties involved describe these shifts as necessary, for instance, when they realize that their decisions as PReMM affect the finances of the entire unit. Furthermore, we argue that using the term collaborative zone is essential, as the more casual word grey zone has a negative connotation. Finally, it is evident that co-leadership “combines both intelligence and sentience” (Gronn and Hamilton, 2004, p. 16).

## Implications for practice

The findings have direct implications for practice across the broad area of healthcare settings where cross-functional leadership between managers and physicians is implemented. Organizations aiming to implement FSL must recognize that it is an emergent, mutual learning process that can scarcely be formalized in advance and certainly not imposed from above.

Sharing leaders themselves benefit from being aware of this shaping process and of the value of both scheduled and informal meetings. To establish a collaborative zone, we emphasize the importance of being physically co-present and allowing mutual learning to unfold over time. We encourage setting aside protected time for mutual reflection to foster learning about oneself, each other and each other’s tasks and responsibilities. We emphasize the importance of not crossing boundaries uninvited, yet invite across boundaries to enable shared responsibility when needed. Finally, we urge sharing leaders to maintain an ongoing dialogue about their shared purpose, as this emerges as the glue of co-leading.

## Conclusions

The shaping of a shared leadership space between physicians responsible for medical management and healthcare managers is an iterative process that is integrated in everyday practice and includes working with boundaries, structures and relationships. Healthcare’s common mission and ways of working facilitated the collaboration despite potential conflicting interests and professional hierarchies. The built-in separation of responsibility and tasks in the FSL form was counteracted as the co-leaders moved toward valuing integrated work in a collaborative zone. Metaphorically the couples perform a co-leadership dance where the steps of either influence the other. The study contributes knowledge relevant for healthcare contexts as well as to the research field of MSL concerning the specific form of FSL.
